# Host-Specific and Segment-Specific Evolutionary Dynamics of Avian and Human Influenza A Viruses: A Systematic Review

**DOI:** 10.1371/journal.pone.0147021

**Published:** 2016-01-13

**Authors:** Kiyeon Kim, Ryosuke Omori, Keisuke Ueno, Sayaka Iida, Kimihito Ito

**Affiliations:** 1 Division of Bioinformatics, Research Center for Zoonosis Control, Hokkaido University, Sapporo, Hokkaido, Japan; 2 PRESTO, Japan Science and Technology Agency (JST), 4-1-8 Honcho, Kawaguchi, Saitama, Japan; 3 Computational Bioscience Research Center, King Abdullah University of Science and Technology, Thuwal, Kingdom of Saudi Arabia; University of Minnesota, UNITED STATES

## Abstract

Understanding the evolutionary dynamics of influenza viruses is essential to control both avian and human influenza. Here, we analyze host-specific and segment-specific Tajima’s D trends of influenza A virus through a systematic review using viral sequences registered in the National Center for Biotechnology Information. To avoid bias from viral population subdivision, viral sequences were stratified according to their sampling locations and sampling years. As a result, we obtained a total of 580 datasets each of which consists of nucleotide sequences of influenza A viruses isolated from a single population of hosts at a single sampling site within a single year. By analyzing nucleotide sequences in the datasets, we found that Tajima’s D values of viral sequences were different depending on hosts and gene segments. Tajima’s D values of viruses isolated from chicken and human samples showed negative, suggesting purifying selection or a rapid population growth of the viruses. The negative Tajima’s D values in rapidly growing viral population were also observed in computer simulations. Tajima’s D values of PB2, PB1, PA, NP, and M genes of the viruses circulating in wild mallards were close to zero, suggesting that these genes have undergone neutral selection in constant-sized population. On the other hand, Tajima’s D values of HA and NA genes of these viruses were positive, indicating HA and NA have undergone balancing selection in wild mallards. Taken together, these results indicated the existence of unknown factors that maintain viral subtypes in wild mallards.

## Introduction

The influenza A virus is a zoonotic pathogen that infects a wide range of mammalian and avian species [1]. According to the antigenicity of hemagglutinin (HA) and neuraminidase (NA), influenza A viruses are divided into 18 HA subtypes and 11 NA subtypes [2]. The natural hosts of influenza A viruses are aquatic birds, such as ducks, geese, and gulls [3]. Sixteen HA subtypes and 9 NA subtypes of influenza A viruses are circulating among these aquatic bird species. So far, H1N1, H2N2, and H3N2 subtype viruses have caused pandemics in humans [4,5]. H5N1, H5N2, and H7N7 subtype viruses cause highly pathogenic avian influenza to chickens, and they have damaged poultry industry for long time [6,7]. Zoonotic transmissions of viruses from pigs and chickens to humans have been reported frequently [8–10].

Since all the influenza A viruses circulating in humans and poultry originated from their natural hosts, understanding the evolutionary dynamics of influenza A viruses in aquatic bird species is important for the control of both avian and human influenza. Kida *et al*. [11] showed ducks infected with influenza A viruses did not show clinical signs of diseases and they produced only low levels of serum antibodies. These results suggested that influenza A viruses have undergone neutral evolution in their natural host population, but clear evidence has yet to be found.

Tajima’s D is a statistic that can be used to test whether or not the population structures of target organisms follow the Wright-Fisher model (WF-model) [12–15]. The WF-model starts from two assumptions. First, the population of target organisms is selectively neutral. Second, the population is constant in size and not subdivided. Using nucleotide sequence data from surveillance studies, Tajima’s D can test whether or not these assumptions hold with the population. Tajima’s D is often used to analyze genetic variation maintained in a population of organisms, including bacteria and viruses [16,17].

In this study, we analyze host-specific and segment-specific Tajima’s D trends of influenza A viruses. To avoid bias from viral population subdivision, we conducted a systematic review of surveillance studies on influenza A viruses of wild mallards, chickens, and humans using nucleotide sequences registered in the database of National Center for Biotechnology Information (NCBI). To our knowledge, this is the first comprehensive Tajima’s D study that uses datasets obtained by stratifying NCBI database sequences according to their isolation hosts, sampling sites, and sampling year. To clarify theoretical detectability of influenza outbreaks by Tajima’s D, we also conducted computer simulations of viral evolution with changing viral demography and confirmed a clear relationship between Tajima’s D and the viral population changes.

## Materials and Methods

### Tajima’s D

Tajima’s D [13] is the normalized difference between two statistics, Watterson’s estimator and Tajima’s estimator. Watterson’s estimator *θ*_*w*_, that is, the expected number of segregating sites between *n* sequences, is given by

$$\theta_{W}=\frac{S_{n}}{\sum_{k=2}^{n}\frac{1}{\left(k-1\right)}}.$$ (1)

The numerator of Eq (1), *S*_*n*_ is the observed number of segregating sites, and the denominator of Eq (1) is the expected total length of genealogy of *n* samples divided by 2 times total population *N*. Tajima’s estimator *θ*_*T*_, which is the average number of nucleotide differences, is given by

$$\theta_{T}=\frac{2}{n(n-1)}\sum_{i<j}\pi_{ij}.$$ (2)

Here *π*_*ij*_ denotes the pairwise difference between the *i*^*th*^ sequence and the *j*^*th*^ sequence in the samples, and *n*(*n*–1)/2 is the total number of pairs in the samples.

Tajima’s D is derived by subtracting Watterson’s estimator from Tajima’s estimator and by normalizing its numerator as follows;

$$D=\frac{\theta_{T}-\theta_{W}}{Std(\theta_{T}-\theta_{W})}.$$ (3)

From Eq (3), the sample size for Tajima’s D have to be larger than three because the denominator of Tajima’s D becomes zero.

### Systematic review

**Fig 1:**
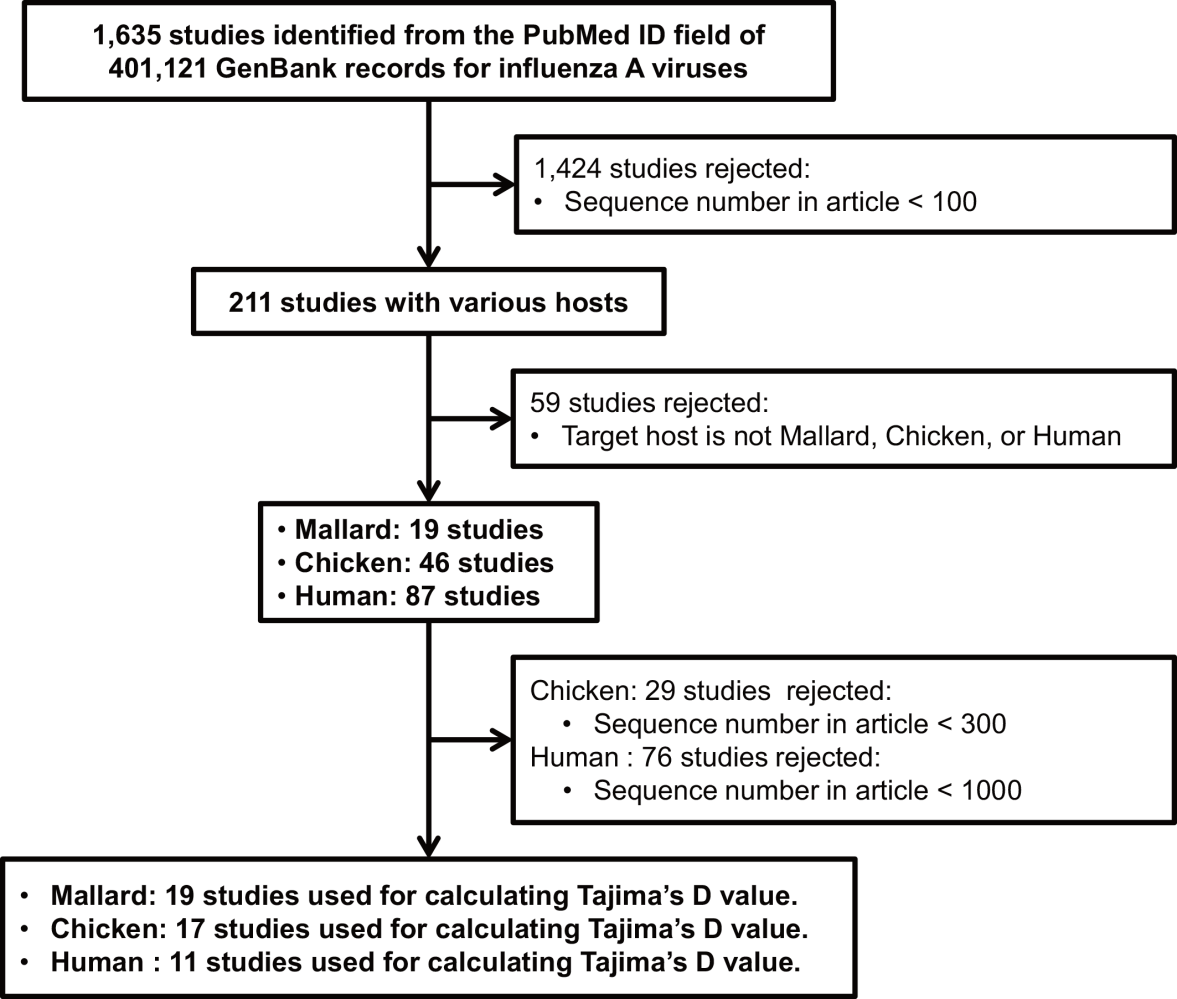
The selection process of systematic review of surveillance studies.

We downloaded all the database records of influenza A viruses from the GenBank on November 24th 2015 by using the Taxonomy ID of influenza viruses as a search condition, i.e. “txid = 11320”. From the retrieved GenBank records, PubMed IDs were collected. Based on the PubMed ID, articles accompanied with more than 100, 300, and 1,000 GenBank sequence records respectively for mallard, chicken, and human viruses were collected. Influenza virus surveillance studies with wild mallards are conducted at a smaller scale than those for chickens and humans. To collect similar numbers of studies, we used these different thresholds on the minim sequence numbers for mallard, chicken, and human. To avoid bias from population subdivision [12,15], the abstract of articles were reviewed, and nucleotide sequences from surveillance studies conducted at a single sampling site from single host species were collected. Fig 1 shows the selection process of the systematic review of surveillance studies.

### Alignment of Sequences and Calculation of Tajima’s D

For each surveillance study selected by above criteria, nucleotide sequences of each gene segment were aligned using MAFFT, a multiple sequence alignment program (version 7) [18]. Sequences with a length less than 90% of complete gene were removed from the alignment. These aligned sequences were stratified according to their sampling years. Since Tajima’s D requires at least four sequences for its calculation, the datasets having less than four sequences were removed. For each dataset containing nucleotide sequences of the same gene segment of influenza A viruses isolated from the same sampling site in a single year, Tajima’s D was computed by a custom program implemented with Python3 (v.3.3.3).

### Outbreak Simulation

Viral sequence evolutions in a rapidly expanding population were simulated using Python3 (python scripts are available in S1 Script and S2 Script). We set the length of nucleotide sequences to 500 and mutation rate in the simulated evolution to 10^−6^ per base per generation. For each generation, viruses are randomly selected from the previous generation with replacement, and their nucleotide sequences were copied to the offspring in the current generation with mutations. We used equal mutation rates for all nucleotide bases (JC69 model) [19]. The simulation was started with 1,000 viruses with identical nucleotide sequences. During the first 5,000 generations, the population size was fixed to 1,000. In each generation from the 5,000^th^ to 5,005^th^, the population size was doubled. From the 5,005^th^ generation, the population size was fixed to 32,000 to the end of the simulation. For every 400 generations, 50 viruses were randomly sampled and Tajima’s D was calculated from their nucleotide sequences. Totally, 100 simulations were conducted with the same setting, and averages of Tajima’s D values were calculated.

## Results

### Data Retrieval and Sequence Alignment

Using 401,212 GenBank records retrieved from the NCBI database, we identified 1,635 articles published with nucleotide sequences of the influenza A viruses. Among them 19, 17, and 11 articles satisfied our criteria for mallard, chicken and human, respectively (Fig 1). A total of 42,664 nucleotide sequences accompanied with these 47 surveillance articles were used calculating Tajima’s D. Table 1 shows the numbers of datasets for each segment and each host after removing dataset having less than four sequences in the alignment. The accession numbers and their nucleotide sequences used in this study can be found in the supplementary information.

**Table 1:** The number of datasets of nucleotide sequences.

Host		PB2	PB1	PA	HA	NP	NA	M	NS
Mallard									
	Number of dataset	24	25	23	24	30	22	29	30
	Total number of sequences	237	240	228	244	367	206	388	315
Chicken									
	Number of dataset	14	19	19	18	18	13	31	23
	Total number of sequences	342	468	414	271	429	143	634	497
Human									
	Number of dataset	24	30	28	16	30	24	33	33
	Total number of sequences	1019	1110	1053	861	1169	880	1191	1191

### Tajima’s D in natural host species

#### Wild Mallard

**Fig 2:**
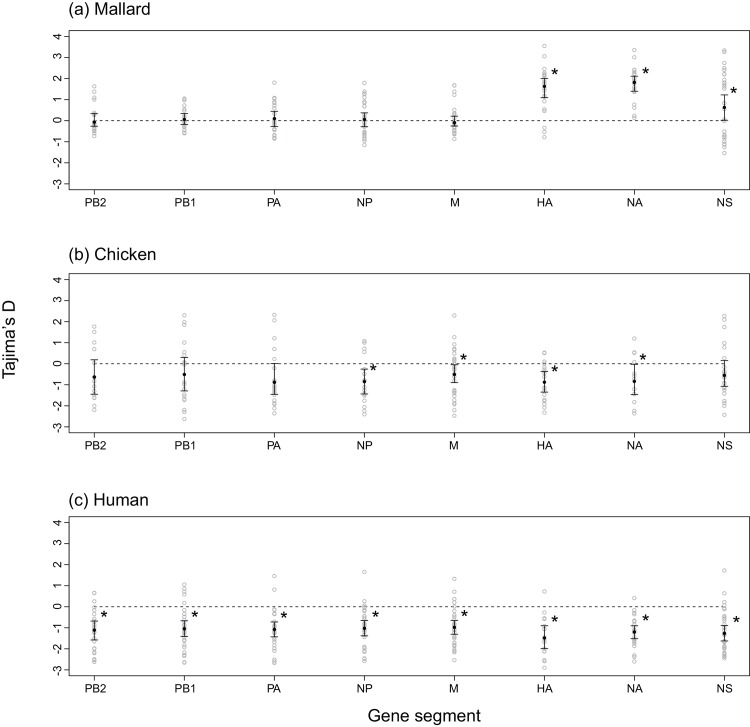
Tajima’s D values for gene segments sampled from the mallards, chickens and humans. (a) shows Tajima's D values for the viruses isolated from wild mallards, (b) shows those from domestic chickens, and (c) shows those from humans. Black circles and error bars represent estimated medians and 95% confidence intervals for the median of Tajima's D across datasets using 1-sample Wilcoxon signed rank test. Gray circles represent Tajima's D values of each dataset. Asterisk denotes the significantly positive or negative Tajima's D based on the result of 1-sample Wilcoxon signed rank test.

The mean of Tajima’s D values of PB2, PB1, PA, NP, and M gene segments were 0.061, 0.028, 0.115, 0.077, and 0.048, respectively (Table 2). Medians of Tajima’s D for the internal gene segments (PB2, PB1, PA, NP, and M) across datasets were close to zero, and the differences from zero were not significant (*p*>0.05, 1-sample Wilcoxon signed rank test) (Fig 2(A)). The mean Tajima’s D of the surface protein genes (HA and NA) and non–structural gene segment (NS) was 1.524, 1.769 and 0.657, respectively (Table 2). Medians of Tajima’s D of these gene segments across datasets were significantly positive (*p*<0.05, 1-sample Wilcoxon signed rank test) (Fig 2(A)).

**Table 2:** The mean and standard deviation of Tajima’s D.

Host		PB2	PB1	PA	HA	NP	NA	M	NS
Mallard									
	Mean	0.061	0.028	0.115	1.524	0.077	1.769	0.048	0.657
	SD	0.630	0.516	0.755	1.065	0.800	0.805	0.672	1.534
Chicken									
	Mean	–0.550	–0.447	–0.678	–0.872	–0.816	–0.766	–0.473	–0.431
	SD	1.325	1.507	1.378	0.903	1.089	1.118	1.100	1.279
Human									
	Mean	–1.109	–1.013	–1.056	–1.417	–1.008	–1.201	–0.941	–1.200
	SD	0.978	1.003	0.940	0.970	0.956	0.730	0.898	0.999

### Tajima’s D in non-natural host species

#### Chicken

Influenza A viruses that were circulating in chickens had an overall mean Tajima D of −0.629. The mean values of Tajima’s D in PB2, PB1, PA, HA, NP, NA, M, and NS gene segments were −0.550, −0.447, −0.678, −0.872, −0.816, −0.766, −0.473, and −0.431, respectively (Table 2). Medians of Tajima’s D values for HA, NP, NA, and MP gene segments across datasets were significantly negative (*p*<0.05, 1-sample Wilcoxon signed rank test) (Fig 2(B)). There were significant differences in Tajima’s D between the wild mallard and chicken except NS gene segment (*p*<0.05; Two-sample Kolmogorov-Smirnov test).

#### Human

Influenza A viruses circulating in humans had a mean Tajima’s D of −1.118. The mean values of Tajima’s D in PB2, PB1, PA, HA, NP, NA, M, and NS gene segments were −1.109, −1.013, −1.056, −1.417, −1.008, −1.201, −0.941, and −1.200, respectively (Table 2). Medians of Tajima’s D for all gene segments were significantly negative (*p*<0.05, 1-sample Wilcoxon signed rank test) (Fig 2(C)), and there were significant differences in Tajima’s D between the wild mallards and humans for all gene segments (*p*<0.05; Two-sample Kolmogorov-Smirnov test). Tajima’s D values for all the dataset can be found in S1 Table.

### Outbreak simulation

**Fig 3:**
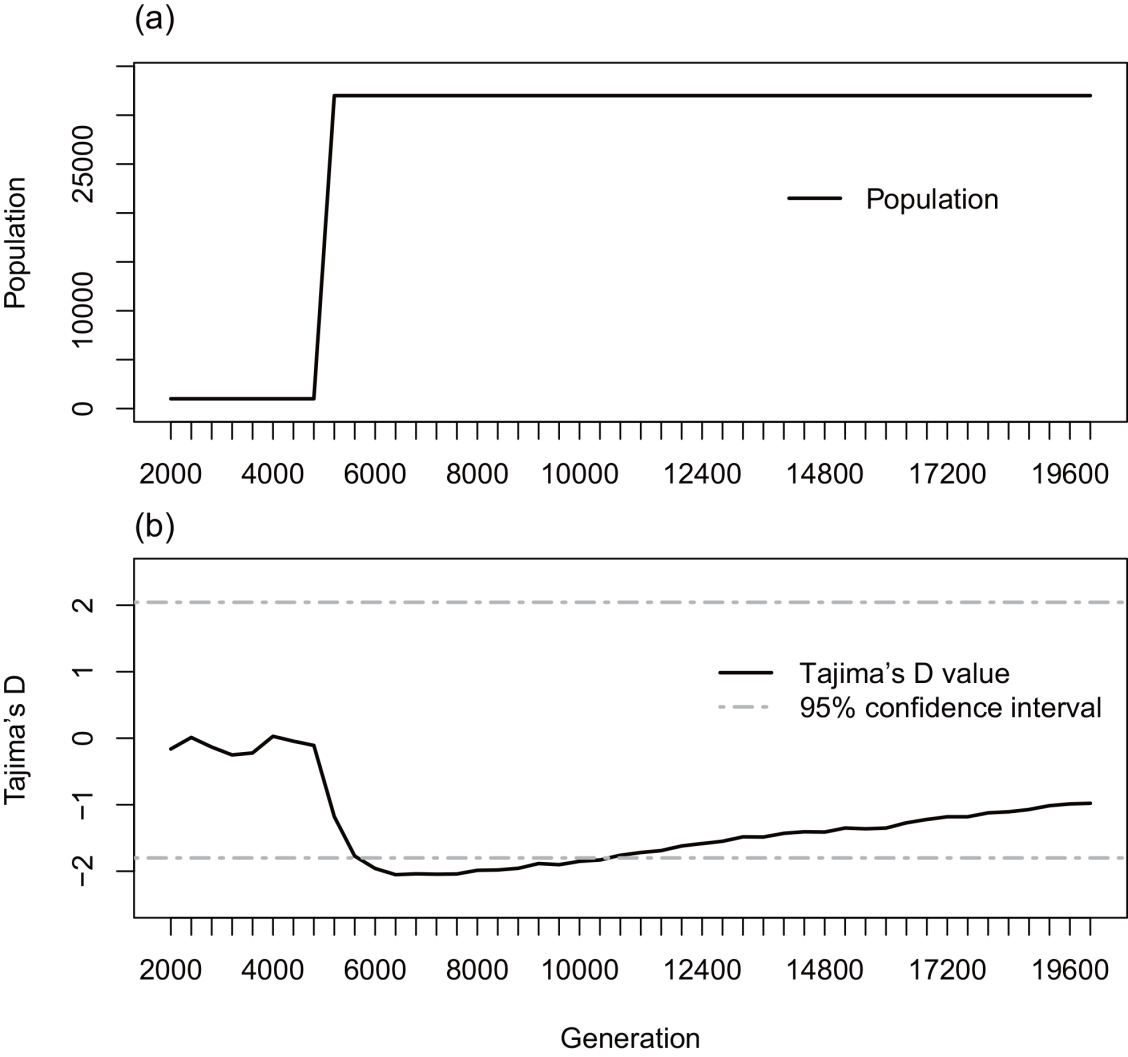
The change of Tajima’s D with a sudden increase of population. (a) shows the setting of time evolution of viral population size and (b) shows the result of time series change of mean Tajima’s D. Gray dot line represents 95% confidence interval of Tajima’s d value for D = 0.

At the first duration when the viral population size was constant over viral generations, the mean of Tajima’s D of 100 simulations was around zero and was within the range of 95% confidence interval for D = 0 (the error distribution was assumed to be beta distribution [13], which agreed with the theory of Tajima’s D. After a sudden increase of the viral population, the mean Tajima’s D value decreased to –2.052, which is significantly negative (*p*<0.05; beta distribution). Consequently the mean Tajima’s D value increased gradually and returned within the range of 95% confidence interval for D = 0 (Fig 3(B)).

## Discussion

In this study, we analyzed host-specific and segment-specific Tajima’s D trends of influenza A viruses through a systematic review of viral sequences registered in the NCBI GenBank. To avoid bias from viral population subdivision, viral sequences were stratified according to their sampling locations and sampling years. Tajima’s D values for internal gene segments of influenza A viruses circulating in wild mallards were close to zero. On the other hand, interestingly, Tajima’s D for external gene segments of influenza A viruses circulating wild mallards showed positive. Tajima’s D values for both internal and external gene segments in non-natural hosts—chicken and human—were negative.

The trends of Tajima’s D are different between internal and external gene segments of influenza A viruses circulating in wild mallards. Wild mallard are considered as the natural host of influenza A viruses. Tajima’s D of influenza viruses in mallards is expected to be close to zero due to the low pathogenicity, or slightly negative due to the selective sweep by low immune response. However, Tajima’s D values for external genes showed positive value, suggesting balancing selection or population subdivision. Since all gene segments should show positive Tajima’s D if viral population were subdivided, balancing selection on external gene segments were more likely to be the cause of positive Tajima’s D values.

To analyze the selection on the external genes of influenza A viruses circulating in wild mallards, we compared Tajima’s D of the data containing only one subtype with those containing multiple subtypes using dataset from Bahl *et al*. [20]. Tajima’s D values of sequences containing two subtypes were positive: the values were 1.159 in 2006 and 1.032 in 2007, suggesting balancing selection. On the other hand, the Tajima’s D for sequences stratified by subtypes were not positive: −0.721 (−1.420) for the H3 HA in wild mallard in 2006 (2007), −1.222 (−0.535) for H4 HA in 2006 (2007), respectively, suggesting neutral or weak purifying selection (Table 3). A similar pattern was observed for NA (Table 4). These results suggested that selection within a subtype was neutral or weak purifying selection as observed in other non-natural hosts, on the other hand, selection across subtypes is balancing selection.

**Table 3:** Subtype specific Tajima’s D of HA in Mallard.

Subtype		Year	
		2006	2007
H3			
	Sample size	11	17
	Tajima’s D	–0.721	–1.42
H4			
	Sample size	6	8
	Tajima’s D	–1.222	–0.535
H3, H4 and others (mixed)			
	Sample size	20	28
	Tajima’s D	1.519	1.032

**Table 4:** Subtype specific Tajima’s D of NA in Mallard.

Subtype		Year	
		2006	2007
N6			
	Sample size	7	9
	Tajima’s D	–0.442	–1.315
N8			
	Sample size	11	14
	Tajima’s D	0.498	–0.011
N6, N8 and others (mixed)			
	Sample size	21	26
	Tajima’s D	2.125	2.052

The diversity of influenza A viruses circulating wild mallard is much higher than other hosts. This high diversity is not able to be explained by relatively low pathogenicity or low immune response of wild mallard, which is one of the main reasons why wild mallard is considered to be the natural host of influenza A viruses. These factors can explain neutral selection on the viruses, but they cannot explain balancing selection.

Several studies have analyzed the evolutionary dynamics of avian influenza viruses using their nucleotide sequences. Time to the most recent common ancestor (TMRCA) of HA, NA and NS were much older than that of internal gene segments [21], and the result is consistent with our results. The phylogenetic analyses of HA and NA suggested high inter-subtype diversity and low intra-subtype diversity, which were not seen in internal gene segments [22]. The distinct divergence between two alleles of NS suggested balancing selection on NS [22], and this was consistent with our results. The dN/dS ratio—the ratio of the number of non-synonymous substitutions per site to the number of synonymous substitution per site—had suggested purifying selection on internal gene segments [20], while Tajima’s D in our study supported neutral selection. This discrepancy between results from dN/dS and Tajima’s D remains as an open question, and one possible explanation for this is that the discrepancy would be attributed to difference between the selection at the lineage level and the selection at the population level.

The negative Tajima’s D values observed in the human and chicken viruses rejected the WF-model for these viral populations. These negative Tajima’s D values should be attributed to the population increase due to recent outbreaks, purifying selection due to viral adaptation to new hosts, or combined effects of population change and selection. However, Tajima’s D itself cannot be used to examine which of these factors are causes of negative Tajima’s D values. This problem highlighted a need for the development of a new methodology that can be used to separate the composite signal into components of population change and selection.

Tajima proposed the use of beta-distribution to reject WF-model and to calculate the 95% confidence interval of Tajima’s D under the WF-model [13]. Our computer simulations showed that Tajima’s D values fell outside 95% confidence interval right after the sudden increase of viral population. Although Simonsen *et al*. [23] showed that criteria using beta-distribution was too conservative to reject WF-model when neutrality assumption does not hold, our computer simulations suggested that beta-distribution could be used to reject WF-model when population size is rapidly growing. When we have multiple samples independently collected form the population, an alternative approach to reject WF-model is to use 1-sample Wilcoxon signed rank test, as shown in the previous section.

It would be of particular interest to find connection between the Tajima’s D of an infectious agent and the effective reproduction number of infectious disease caused by the agent. The effective reproduction number measures the continuance of an outbreak and the expected number of secondary infections. Recent studies have utilized coalescent theory to estimate the time evolution of population size of the ancestors of sampled sequences. By assuming constant-sized population between two coalescence events, Pybus *et al*. developed a method to estimate the time evolution of population size from their nucleotide sequences [24]. Mathematical models on population dynamics of infectious diseases have been also proposed to characterize infectious disease outbreaks from nucleotide sequences of infectious agents [25,26].

## Conclusions

We calculated host-specific and segment specific Tajima’s D values of influenza A viruses through a systematic review using viral sequences registered in the NCBI database. Interestingly, sequences encoding external proteins of influenza A viruses showed positive Tajima’s D in wild mallards, suggesting the existence of balancing selection, although zero or negative Tajima’s D was expected. This result suggests the existence of missing factors other than low immune response or low pathogenicity to maintain the variation of the subtypes circulating in the natural hosts.

## Supporting Information

S1 PRISMA Checklist (DOC)

S1 Dataset Accession numbers in fasta format that are used in this study. (ZIP)

S1 Script Python3 code for outbreak simulation. (PY)

S2 Script Python3 code for calculating Tajima’s D value. (PY)

S1 Table Variables—number of sequences, length of sequences, Watterson’s estimator and Tajima’s estimator, Tajima's D value, and 95% confidence interval for each data set. ^a^Watterson’s estimator. ^b^Tajima’s estimator. ^c^Tajima’s D value.
(XLSX)
